# A systematic review and meta-analysis of regional risk factors for critical outcomes of COVID-19 during early phase of the pandemic

**DOI:** 10.1038/s41598-021-89182-8

**Published:** 2021-05-07

**Authors:** Hyung-Jun Kim, Hyeontaek Hwang, Hyunsook Hong, Jae-Joon Yim, Jinwoo Lee

**Affiliations:** 1 Division of Pulmonary and Critical Care Medicine, Department of Internal Medicine, Armed Forces Capital Hospital, Seongnam, Republic of Korea; 2 Division of Pulmonary and Critical Care Medicine, Department of Internal Medicine, Seoul National University Hospital, Seoul, Republic of Korea; 3 Department of Internal Medicine, Seoul National University College of Medicine, 103 Daehak-Ro, Jongno-Gu, Seoul, 03080 Republic of Korea; 4 Division of Medical Statistics, Medical Research Collaborating Center, Seoul National University Hospital, Seoul, Republic of Korea

**Keywords:** Risk factors, Signs and symptoms, Prognosis, Medical research, Outcomes research

## Abstract

The mortality rates of COVID-19 vary across the globe. While some risk factors for poor prognosis of the disease are known, regional differences are suspected. We reviewed the risk factors for critical outcomes of COVID-19 according to the location of the infected patients, from various literature databases from January 1 through June 8, 2020. Candidate variables to predict the outcome included patient demographics, underlying medical conditions, symptoms, and laboratory findings. The risk factors in the overall population included sex, age, and all inspected underlying medical conditions. Symptoms of dyspnea, anorexia, dizziness, fatigue, and certain laboratory findings were also indicators of the critical outcome. Underlying respiratory disease was associated higher risk of the critical outcome in studies from Asia and Europe, but not North America. Underlying hepatic disease was associated with a higher risk of the critical outcome from Europe, but not from Asia and North America. Symptoms of vomiting, anorexia, dizziness, and fatigue were significantly associated with the critical outcome in studies from Asia, but not from Europe and North America. Hemoglobin and platelet count affected patients differently in Asia compared to those in Europe and North America. Such regional discrepancies should be considered when treating patients with COVID-19.

## Introduction

Coronavirus disease 2019 (COVID-19) is an acute respiratory illness caused by the novel severe acute respiratory syndrome virus 2, which was first reported in Wuhan, China^1,2^. Because the virus is highly contagious, it has caused a global pandemic^3^. Disparities exist across the globe. According to the World Health Organization Dashboard as of December 10, 2020^4^, 29.1 million cases were confirmed with 760.9 thousand deaths in the Americas, and 20.9 million cases with 462.6 thousand deaths in Europe, and 11.2 million confirmed cases and 170.9 thousand deaths in South-East Asia. This corresponds to calculated mortality rates of 2.61%, 2.22%, and 1.52%, respectively.

The COVID-19 outbreak has rapidly overloaded healthcare facilities^5^. Since the availability of these resources is crucial for patient survival, areas with sudden upsurges in patients showed higher mortality rates^6,7^. Recognizing the patient-at-risk characteristics is important for the distribution of patients to appropriate levels of care. In addition, prioritizing people as candidates for potential vaccines is also necessary^8^.

The risk factors for poor outcomes of COVID-19 have been reviewed. A previous systematic review of 13 studies reported male sex, older age, smoking, underlying comorbidities, symptoms of dyspnea, and several laboratory findings as significant factors for poor prognoses^9^. Another review including 14 studies reported similar results^10^. However, most of the included studies in these meta-analyses were from China because COVID-19 was mostly spread in China during the early phase of the pandemic. Summarized results from other parts of the world have not yet been reported.

This study systematically reviewed the differences in risk factors for critical outcomes of patients with COVID-19 according to the continent on which the studies were performed. In addition, we aimed to update the risk factors based on a wider range of studies.

## Methods

### Search strategy and study protocol

We searched PubMed, Embase, Cochrane Library, and Web of Science literature databases using keywords related to COVID-19, hospitalized adult patients, and critical outcomes to identify studies published from January 1 to June 8, 2020. The critical outcome was defined as death, admission to the intensive care unit (ICU), or critical type of COVID-19. The critical type of COVID-19 was defined as COVID-19 with respiratory failure, septic shock, or multiple organ dysfunction^11^. We followed the Peer Review of Electronic Search Strategies to design a structural search strategy (see Supplementary Data S1)^12^. We also conducted a manual search using study identifiers or references from previous studies.

Our systematic review was performed according to the Preferred Reporting Items for Systematic Reviews and Meta-Analysis (PRISMA) guidelines^13^ and Meta-analysis of Observational Studies in Epidemiology^14^. The PRISMA checklist is available from Supplementary Data S2, and the protocol for this systematic review was registered on International Prospective Register of Systematic Reviews (www.crd.york.ac.uk/PROSPERO/display_record.asp?ID=CRD42020181062).

### Study selection

Studies were selected by following the PRISMA flow diagram^13^. After removing duplicates, the titles and abstracts were screened to identify eligible studies for full-text review. When different outcome data were found in the same study population with similar study periods, the data with the larger population were selected. Studies with ≤ 5 patients with critical outcomes were excluded because the calculations of mean and standard deviations (SDs) were considered unreliable in these studies. When data were not presented according to the critical outcome, the authors were contacted to provide organized results. Studies performed in the ICU, or those including patients with negative COVID-19 polymerase chain reaction results were also excluded.

### Data extraction

From each study, we collected article information including the authors, study design, location, period, restriction in patient selection, and study outcome. Patient characteristics were collected including sex, age, body mass index (BMI), ethnicity, underlying medical condition, symptoms, and laboratory findings. The underlying medical condition included smoking history, hypertension, diabetes, cardiac disease, renal disease, respiratory disease, hepatic disease, cerebral disease, malignancy. The comorbidities were defined differently in each study, as shown in Supplementary Table S1. The symptoms included fever, fatigue, myalgia, dizziness, headache, dyspnea, chest tightness, cough, sputum, sore throat, rhinorrhea, anorexia, nausea, vomiting, abdominal pain, and diarrhea. The laboratory findings included white blood cell count, neutrophil count, lymphocyte count, monocyte count, hemoglobin, platelet count, creatinine, blood urea nitrogen, aspartate transaminase (AST), alanine transaminase (ALT), total bilirubin, creatine kinase, lactate dehydrogenase, prothrombin time, D-dimer, troponin (troponin I or T), and *pro* brain-type natriuretic peptide (proBNP).

The characteristics were organized according to the critical outcome defined in each study. For categorical variables, the input variables were organized as a two-by-two table. For continuous variables, means and SDs were organized as recommended by the Cochrane handbook^15^. Google Translate was used to translate the articles published in Chinese to English.

### Statistical considerations and assessment of bias

Forest plots with a random-effects model were used to explore the baseline characteristics and the impact of each variable on the critical outcome. I^2^ statistics were used to assess the heterogeneity^16^. Pooled relative risks (RRs) were calculated for categorical variables. For continuous variables, standardized mean differences (SMDs) were calculated for most variables because of the differences in scale, except for age and BMI for which weighted mean difference (WMD) was calculated. The 95% confidence intervals were calculated for each pooled value and are presented in square brackets throughout the manuscript.

Quality assessment of each study was performed according to the recommended six areas of potential study biases: study participation, study attrition, outcome measurement, confounding measurement and account, and analysis^17^. Egger’s regression tests were performed to assess publication bias^18^.

Analyses were performed in the overall population and in subgroups according to the continent. The impact of ethnicity on the critical outcome was inspected separately with studies specifying the race according to the four categories: non-Hispanic white, non-Hispanic black, Hispanic, and Asian. To reduce the heterogeneity of the results, sensitivity analyses were performed among studies without any restriction in patient selection, critical outcome confined to death, and at least partly achieving every standard of the six areas of potential study biases.

The process of study screening, data extraction, and assessment of quality and risk of bias were performed by two independent reviewers, and an agreement was reached through group discussion. All statistical analyses were performed using Stata version 16 (StataCorp. 2019. Stata Statistical Software: Release 16. College Station, TX, StataCorp LLC).

## Results

### Search findings and study characteristics

The initial search revealed 3071 studies, which narrowed to 2151 studies after duplicate removal. After screening, 1578 studies were removed and 573 articles were assessed with full-text review. After removing 493 non-relevant studies, our systematic review included a total of 80 studies (Supplementary Fig. S1). The full list of the included studies is available in Supplementary Data S3^19–98^.

The 80 studies included 43,248 patients, with a median of 130 patients per study (interquartile range 73–317). The studies were conducted in Asia (n = 48), Europe (n = 22), and North America (n = 10). The countries included China (n = 43), the United States of America (n = 10), Italy (n = 9), Spain (n = 5), Iran (n = 3), the United Kingdom (n = 3), South Korea (n = 1), India (n = 1), Denmark (n = 1), France (n = 1), Greece (n = 1), Norway (n = 1), and Poland (n = 1). The study outcomes were death (n = 41, 51.3%), admission to the ICU (n = 15, 18.8%), admission to the ICU or death (n = 9, 11.3%), and critical type of COVID-19 (n = 15, 18.8%). A median of 23.7% of patients had suffered the critical outcome in the overall population and was highest in studies from North America (34.8%), followed by Europe (26.5%) and Asia (17.3%) (*P* < 0.001). Most of the studies were retrospective observational studies (n = 68, 85.0%). Of the 80 studies, 50 (62.5%) did not specify any restriction in patient selection, while 13 studies included patients with certain comorbidities, seven with certain COVID-19 severity, and four with computed tomography findings (Table 1).

**Table 1:** Characteristics of studies included in analysis.

Variables		Total	Asia	Europe	North America	*P*
		N = 80	n = 48	n = 22	n = 10	
Number of patients		130 (73–317)	136 (99–323)	114 (36–233)	104 (72–1000)	0.328
**Study outcome**						< 0.001
Death		41 (51.3)	24 (50.0)	15 (68.2)	2 (20.0)	
Admission to the ICU		15 (18.8)	6 (12.5)	5 (22.7)	4 (40.0)	
Admission to the ICU or death		9 (11.3)	3 (6.3)	2 (9.1)	4 (40.0)	
Critical type COVID-19*		15 (18.8)	15 (31.3)	0 (0.0)	0 (0.0)	
Proportion of patients with critical outcome, %		23.7 (14.8–34.4)	17.3 (13.6–27.9)	26.4 (19.0–45.8)	34.8 (30.0–42.7)	< 0.001
**Country**						NA
China		43 (53.8)	43 (89.6)	0	0	
United States of America		10 (12.5)	0	0	10 (100.0)	
Italy		9 (11.3)	0	9 (40.9)	0	
Spain		5 (6.3)	0	5 (22.7)	0	
Iran		3 (3.8)	3 (6.3)	0	0	
United Kingdom		3 (3.8)	0	3 (13.6)	0	
South Korea		1 (1.3)	1 (2.1)	0	0	
India		1 (1.3)	1 (2.1)	0	0	
Denmark		1 (1.3)	0	1 (4.6)	0	
France		1 (1.3)	0	1 (4.6)	0	
Greece		1 (1.3)	0	1 (4.6)	0	
Norway		1 (1.3)	0	1 (4.6)	0	
Poland		1 (1.3)	0	1 (4.6)	0	
**Study design**						0.004
Retrospective observational		68 (85.0)	45 (93.8)	14 (63.6)	9 (90.9)	
Prospective cohort		12 (15.0)	3 (6.3)	8 (36.4)	1 (10.0)	
**Restriction in patient selection**						0.856
None		50 (62.5)	30 (62.5)	12 (54.6)	8 (80.0)	
Certain comorbidity		13 (16.3)	6 (12.5)	5 (22.7)	2 (20.0)	
Certain severity		7 (8.8)	5 (10.4)	2 (9.1)	0 (0.0)	
Patients with CT results		4 (5.0)	3 (6.3)	1 (4.6)	0 (0.0)	
Certain age group		2 (2.5)	2 (4.2)	0 (0.0)	0 (0.0)	
Certain symptom		1 (1.3)	1 (2.1)	0 (0.0)	0 (0.0)	
Multiple PCR tests		1 (1.3)	1 (2.1)	0 (0.0)	0 (0.0)	
Hospitalized via ER		1 (1.3)	0 (0.0)	1 (4.6)	0 (0.0)	
Certain race		1 (1.3)	0 (0.0)	1 (4.6)	0 (0.0)	

Numbers are presented as number (percentage) or median (interquartile range). *P*-values are calculated from chi-square test, Fisher’s exact test, or Kruskal–Wallis test.

*Critical type COVID-19 refers to disease extent with respiratory failure, septic shock, and/or multiple organ dysfunction.

*ICU* intensive care unit, *NA* not applicable, *CT* computed tomography, *PCR* polymerase chain reaction, *ER* emergency room.

### Baseline patient characteristics and symptoms

In the overall population, a proportion of 0.57 [0.55–0.59] were male, with a mean age of 68.5 [65.1–71.8] years, and BMI of 26.5 [23.2–29.8] kg/m^2^. The common underlying medical conditions were hypertension (pooled proportion 0.41 [0.35–0.47]), smoking history (pooled proportion 0.23 [0.19–0.27]), and diabetes (pooled proportion 0.21 [0.17–0.25]). The common symptoms were fever (pooled proportion 0.79 [0.70–0.86]), cough (pooled proportion 0.65 [0.60–0.70]), and anorexia (pooled proportion 0.58 [0.43–0.72]) (Table 2).

**Table 2:** Summary of patient characteristics according to the continents of the studies performed.

Variables		Total	n	Asia	n	Europe	n	North America	n
Male sex		0.57 (0.55–0.59)	51	0.52 (0.50–0.55)	24	0.64 (0.61–0.68)	19	0.57 (0.53–0.60)	8
Age, years		68.5 (65.1–71.8)	52	58.6 (53.0–64.2)	25	75.4 (70.9–79.8)	19	63.5 (51.4–75.6)	8
Body mass index, kg/m^2^		26.5 (23.2–29.8)	8	22.0 (14.7–29.3)	1	27.2 (23.0–31.5)	4	29.2 (21.6–36.8)	3
**Ethnicity**									
Non-Hispanic White		0.30 (0.13–0.47)	4	–	0	–	0	0.30 (0.13–0.47)	4
Hispanic		0.27 (0.24–0.29)	4	–	0	–	0	0.27 (0.24–0.29)	4
Non-Hispanic black		0.15 (0.10–0.19)	4	–	0	–	0	0.15 (0.10–0.19)	4
Asian		0.06 (0.02–0.10)	4	–	0	–	0	0.06 (0.02–0.10)	4
Unknown/Others		0.21 (0.00–0.42)	4	–	0	–	0	0.21 (0.00–0.42)	4
**Underlying medical condition**									
Hypertension		0.41 (0.35–0.47)	45	0.27 (0.23–0.31)	21	0.51 (0.44–0.59)	16	0.62 (0.58–0.66)	8
Smoking history		0.23 (0.19–0.27)	23	0.13 (0.04–0.26)	8	0.29 (0.21–0.39)	8	0.30 (0.25–0.35)	7
Diabetes		0.21 (0.17–0.25)	46	0.15 (0.12–0.17)	20	0.21 (0.17–0.24)	18	0.38 (0.32–0.43)	8
Cardiac disease		0.18 (0.15–0.22)	46	0.13 (0.08–0.20)	21	0.24 (0.18–0.31)	17	0.20 (0.16–0.24)	8
Renal disease		0.12 (0.09–0.15)	33	0.04 (0.02–0.06)	11	0.18 (0.12–0.25)	14	0.21 (0.16–0.27)	8
Malignancy		0.10 (0.08–0.12)	38	0.03 (0.02–0.04)	16	0.23 (0.16–0.32)	15	0.11 (0.08–0.15)	7
Respiratory disease		0.09 (0.07–0.11)	44	0.04 (0.03–0.06)	20	0.12 (0.10–0.15)	17	0.17 (0.13–0.21)	7
Hepatic disease		0.04 (0.02–0.06)	18	0.06 (0.01–0.12)	13	0.01 (0.01–0.01)	3	0.01 (0.01–0.02)	2
Cerebral disease		0.06 (0.05–0.08)	22	0.05 (0.03–0.07)	12	0.09 (0.06–0.11)	7	0.06 (0.02–0.11)	3
**Symptoms**									
Fever		0.79 (0.70–0.86)	32	0.82 (0.76–0.88)	17	0.80 (0.64–0.92)	9	0.65 (0.42–0.85)	6
Cough		0.65 (0.60–0.70)	31	0.66 (0.59–0.73)	17	0.60 (0.50–0.70)	10	0.71 (0.63–0.79)	4
Anorexia		0.58 (0.43–0.72)	8	0.62 (0.47–0.75)	7	–	0	0.31 (0.23–0.41)	1
Fatigue		0.44 (0.32–0.55)	17	0.50 (0.34–0.67)	10	0.26 (0.17–0.37)	3	0.39 (0.28–0.51)	4
Dyspnea		0.43 (0.34–0.52)	28	0.31 (0.21–0.42)	14	0.50 (0.39–0.60)	9	0.64 (0.57–0.70)	5
Sputum		0.27 (0.20–0.35)	16	0.35 (0.30–0.40)	10	0.16 (0.08–0.27)	3	0.15 (0.06–0.25)	3
Myalgia		0.22 (0.17–0.27)	22	0.22 (0.16–0.30)	12	0.17 (0.09–0.28)	6	0.30 (0.22–0.38)	4
Chest tightness		0.21 (0.11–0.33)	10	0.26 (0.13–0.41)	7	–	0	0.12 (0.07–0.17)	3
Dizziness		0.14 (0.04–0.28)	5	0.13 (0.03–0.28)	4	–	0	0.19 (0.04–0.46)	1
Diarrhea		0.14 (0.10–0.19)	25	0.11 (0.10–0.19)	14	0.17 (0.09–0.28)	6	0.23 (0.21–0.26)	5
Headache		0.12 (0.08–0.16)	19	0.10 (0.04–0.17)	10	0.17 (0.05–0.32)	4	0.12 (0.08–0.16)	5
Nausea		0.12 (0.07–0.17)	12	0.08 (0.04–0.13)	5	0.09 (0.02–0.21)	3	0.18 (0.16–0.20)	4
Sore throat		0.09 (0.06–0.12)	15	0.09 (0.05–0.14)	8	0.09 (0.02–0.19)	3	0.08 (0.06–0.10)	4
Vomiting		0.08 (0.02–0.18)	7	0.08 (0.01–0.22)	5	–	0	0.09 (0.05–0.14)	2
Rhinorrhea		0.08 (0.04–0.12)	5	0.04 (0.02–0.07)	2	–	0	0.10 (0.06–0.14)	3
Abdominal pain		0.04 (0.01–0.09)	5	0.04 (0.01–0.09)	5	–	0	–	0

Numbers are presented as pooled value with 95% confidence intervals. Values represent proportions unless specified otherwise.

*n* numbers of studies included in calculating the pooled value in the previous column.

### Impacts of baseline demographics and underlying medical condition on the critical outcome

Of the 43,248 patients, 10,652 suffered the critical outcome. A meta-analysis of 51 studies revealed that male sex was associated with an increased risk of the critical outcome (pooled RR 1.26 [1.17–1.36], I^2^ = 36.7%). The results remained consistent in subgroup analyses of each continent: 24 studies from Asia showed a pooled RR of 1.42 [1.26–1.61] (I^2^ = 0.0%), 19 studies from Europe showed a pooled RR of 1.19 [1.02–1.40] (I^2^ = 48.2%), and 8 studies from North America showed a pooled RR of 1.23 [1.07–1.42] (I^2^ = 61.2%) (Supplementary Fig. S2). Older age was associated with an increased risk of the critical outcome in the overall analysis (pooled WMD 8.69 [7.22–10.16], I^2^ = 89.9%) and remained significant in the subgroup analysis of studies from Asia (pooled WMD 11.23 [8.63–13.83], I^2^ = 86.7%, 24 studies) and Europe (pooled WMD 7.65 [4.97–10.33], I^2^ = 86.5%, 17 studies) (Supplementary Fig. S3). BMI was not associated with the increased risk of the critical outcome in the overall analysis (pooled WMD 1.03 [− 0.12–2.17], I^2^ = 77.1%, 8 studies). The association between higher BMI and the critical outcome was significant in one study from Asia (WMD 3.50 [1.76–5.24]) but not in those from Europe (pooled WMD 0.83 [− 1.21–2.87], I^2^ = 51.3%, 4 studies) and North America (pooled WMD 0.40 [− 0.71–1.50], I^2^ = 75.3%, 3 studies).

**Figure 1:**
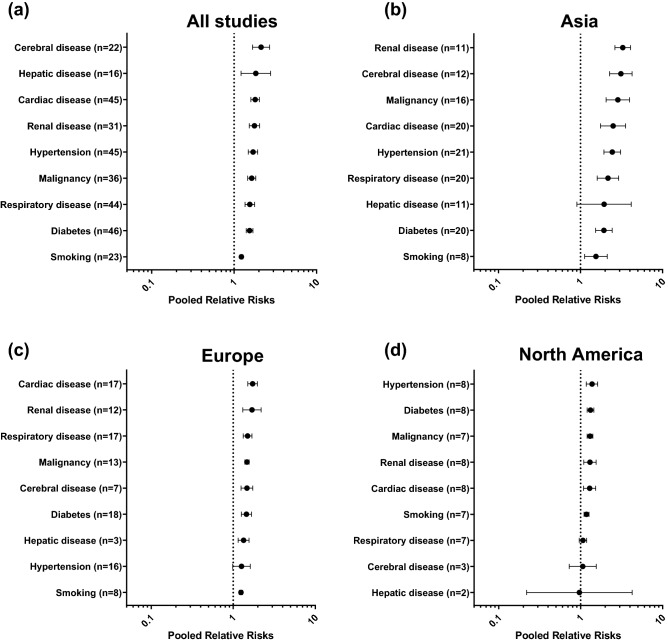
Impacts of underlying medical conditions on the critical outcome of COVID-19. The numbers in parenthesis represent the number of studies included in the pooled analysis. (**a**) Pooled analysis of all included studies. (**b**)–(**d**) Pooled analyses of studies performed in Asia, Europe, and North America, respectively.

Underlying medical condition including cerebral disease (pooled RR 2.12 [1.67–2.70], I^2^ = 82.8%, 22 studies), hepatic disease (pooled RR 1.84 [1.22–2.77], I^2^ = 64.5%, 16 studies), cardiac disease (pooled RR 1.80 [1.60–2.03], I^2^ = 74.7%, 45 studies), renal disease (pooled RR 1.76 [1.53–2.04], I^2^ = 78.0%, 31 studies), hypertension (pooled RR 1.70 [1.49–1.93], I^2^ = 71.2%, 45 studies), malignancy (pooled RR 1.64 [1.46–1.83], I^2^ = 51.6%, 36 studies), respiratory disease (pooled RR 1.55 [1.36–1.78], I^2^ = 64.9%, 44 studies), diabetes (pooled RR 1.54 [1.40–1.69], I^2^ = 58.0%, 46 studies), and smoking history (pooled RR 1.23 [1.18–1.28], I^2^ = 0.1%, 23 studies) were risk factors for the critical outcome. Subgroup analyses across the three continents showed largely similar results; however, several differences were noted. First, the presence of respiratory disease was associated with a higher risk of the critical outcome in studies from Asia (pooled RR 2.16 [1.60–2.92], I^2^ = 57.8%, 20 studies) and Europe (pooled RR 1.50 [1.32–1.69], I^2^ = 16.6%, 17 studies), but not North America (pooled RR 1.07 [0.96–1.19], I^2^ = 0.0%, 7 studies). Second, the presence of hepatic disease was associated with a higher risk of the critical outcome from Europe (pooled RR 1.34 [1.15–1.56], I^2^ = 0.0%, 3 studies), but not from Asia (pooled RR 1.94 [0.90–4.16], I^2^ = 68.1%, 11 studies) and North America (pooled RR 0.97 [0.22–4.25], I^2^ = 39.4%, 2 studies) (Fig. 1).

### Associations between patient symptoms and the critical outcome

**Figure2:**
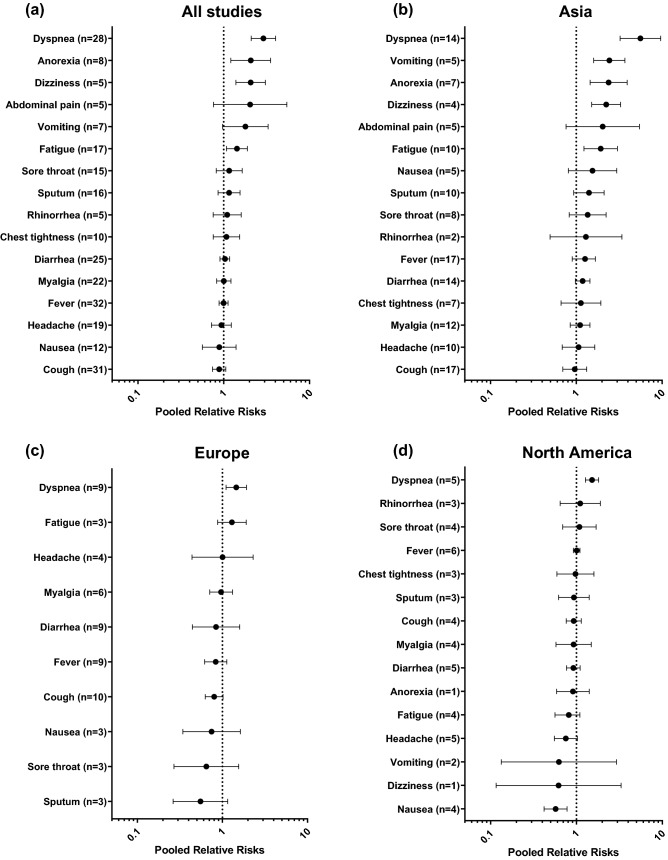
Impacts of patient symptoms on the critical outcome of COVID-19. The numbers in parenthesis represent the number of studies included in the pooled analysis. (**a**) Pooled analysis of all included studies. (**b**)–(**d**) Pooled analyses of studies performed in Asia, Europe, and North America, respectively.

The results of the meta-analysis showed that dyspnea (pooled RR 2.90 [2.10–4.03], I^2^ = 85.7%, 28 studies), anorexia (pooled RR 2.07 [1.21–3.52], I^2^ = 69.8%, 8 studies), dizziness (pooled RR 2.06 [1.39–3.06], I^2^ = 5.1%, 5 studies), and fatigue (pooled RR 1.43 [1.08–1.89], I^2^ = 62.8%, 17 studies) were significantly associated with the critical outcome. In the subgroup analysis, dyspnea was the only symptom that was consistently associated with the poor outcome in Asia (pooled RR 5.60 [3.24–9.65], I^2^ = 84.0%, 14 studies), Europe (pooled RR 1.45 [1.10–1.91], I^2^ = 28.7%, 9 studies), and North America (pooled RR 1.52 [1.27–1.81], I^2^ = 0.0%, 5 studies). Vomiting (pooled RR 2.43 [1.60–3.69], I^2^ = 0.0%, 5 studies), anorexia (pooled RR 2.38 [1.45–3.91], I^2^ = 49.5%, 7 studies), dizziness (pooled RR 2.23 [1.51–3.28], I^2^ = 0.0%, 4 studies), and fatigue (pooled RR 1.92 [1.23–3.02], I^2^ = 72.3%, 10 studies) were significantly associated with the critical outcome in studies from Asia, but not from Europe and North America (Fig. 2).

### Associations between laboratory findings and the critical outcome

Higher levels of proBNP (pooled SMD 1.42 [0.52–2.31], I^2^ = 97.6%, 5 studies), lactate dehydrogenase (pooled SMD 1.28 [1.02–1.54], I^2^ = 91.0%, 23 studies), blood urea nitrogen (pooled SMD 1.04 [0.62–1.45], I^2^ = 93.6%, 15 studies), neutrophil count (pooled SMD 0.92 [0.61–1.22], I^2^ = 92.7%, 28 studies), AST (pooled SMD 0.78 [0.61–0.96], I^2^ = 87.4%, 26 studies), white blood cell count (pooled SMD 0.75 [0.51–1.00], I^2^ = 92.6%, 34 studies), troponin (pooled SMD 0.67 [0.36–0.98], I^2^ = 94.7%, 14 studies), D-dimer (pooled SMD 0.57 [0.36–0.78], I^2^ = 92.2%, 25 studies), creatine kinase (pooled SMD 0.53 [0.23–0.84], I^2^ = 89.5%, 18 studies), creatinine (pooled SMD 0.51 [0.36–0.66], I^2^ = 84.3%, 32 studies), prothrombin time (pooled SMD 0.44 [0.31–0.58], I^2^ = 32.2%, 12 studies), total bilirubin (pooled SMD 0.36 [0.22–0.51], I^2^ = 54.8%, 19 studies), and ALT (pooled SMD 0.26 [0.18–0.33], I^2^ = 30.2%, 30 studies) were associated with the critical outcome. In contrast, levels of hemoglobin (pooled SMD − 0.21 [− 0.37 to − 0.05], I^2^ = 67.8%, 26 studies), platelet count (pooled SMD − 0.21 [− 0.36 to − 0.06], I^2^ = 72.5%, 31 studies), and lymphocyte count (pooled SMD − 0.58 [− 0.71 to − 0.45], I^2^ = 76.8%, 37 studies) were inversely related to the critical outcome.

**Figure 3:**
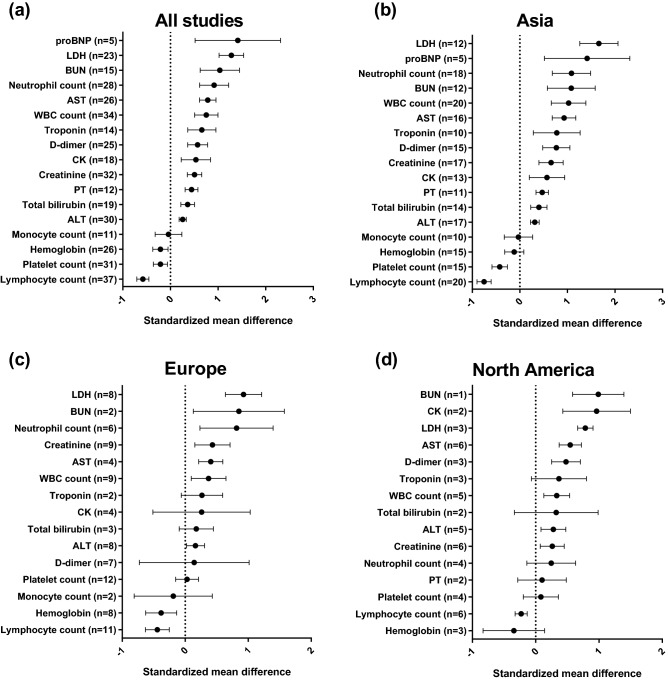
Associations between laboratory findings and the critical outcome of COVID-19. The numbers in parenthesis represent the number of studies included in the pooled analysis. (**a**) Pooled analysis of all included studies. (**b**)–(**d**) Pooled analyses of studies performed in Asia, Europe, and North America, respectively.

While many findings were consistent across the three continents, platelet count and hemoglobin levels showed different results. While platelet count was inversely associated with the critical outcome in studies from Asia (pooled SMD − 0.42 [− 0.59 to − 0.26], I^2^ = 62.7%, 15 studies), this association was not observed in studies from Europe (pooled SMD 0.03 [− 0.15 to 0.21], I^2^ = 39.5%, 12 studies) or North America (pooled SMD 0.08 [0.19–0.36], I^2^ = 16.2%, 4 studies). In contrast, while lower hemoglobin levels were associated with the critical outcome in studies from Europe (pooled SMD − 0.38 [− 0.63 to − 0.14], I^2^ = 34.5%, 8 studies), this association was not identified in studies from Asia (pooled SMD − 0.12 [− 0.32 to 0.08], I^2^ = 72.0%, 15 studies) or North America (pooled SMD − 0.35 [− 0.83 to 0.14], I^2^ = 57.6%, 3 studies) (Fig. 3).

### Impact of ethnicity on the critical outcome

Out of a total of 80 studies, only four studies from North America reported patients’ ethnicity according to the four categories (non-Hispanic white, non-Hispanic black, Hispanic, and Asian). The pooled proportions of each ethnicity were non-Hispanic white (0.30 [0.13–0.47]), Hispanic (0.27 [0.24–0.29]), non-Hispanic black (0.15 [0.10–0.19]), Asian (0.06 [0.02–0.10]), and others/unknown (0.21 [0.00–0.42]) (Table 2).

Compared with non-Hispanic white, Hispanic ethnicity (pooled RR 0.83 [0.71–0.96], I^2^ = 8.4%) was associated with a lower risk of the critical outcome, while non-Hispanic black (pooled RR 0.84 [0.67–1.06], I^2^ = 28.3%) and Asian ethnicity (pooled RR 1.33 [0.86–2.06], I^2^ = 51.8%) was not (Supplementary Fig. S4). Publication biases were not observed in these analyses (Egger’s *P* = 0.388, 0.282, and 0.557, respectively).

### Assessment of study quality and publication bias

While most studies at least partly met the quality standards of each area, several studies did not. The two studies did not represent the population of interest (study participation), two studies did not adequately measure the prognostic factor of interest (prognostic factor measurement), and nine studies did not account for important potential confounders (confounding measurement and account). (Supplementary Table S2).

Most of the variables did not show any publication bias; however, male sex (*P* = 0.042), underlying diabetes (*P* = 0.001), malignancy (*P* = 0.020), cerebral disease (*P* = 0.042), symptoms of dyspnea (*P* = 0.019), and vomiting (*P* = 0.045) revealed significant publication bias. Laboratory findings of lymphocyte count (*P* = 0.003), ALT (*P* = 0.012), and AST (*P* = 0.023) also revealed publication biases (Supplementary Table S3).

### Sensitivity analysis

A sensitivity analysis was performed in 17 studies without any restriction in patient selection, outcome confined to death, and at least partly achieving every standard of the six areas of potential study biases. The results were largely consistent with the main analyses. Male sex (pooled RR 1.17 [1.02–1.34], I^2^ = 44.8%, 15 studies) was a significant risk factor. The sensitivity analysis revealed older age to be a risk factor for death in all three continents (pooled WMD 12.44 [10.76–14.13], I^2^ = 79.0%, 14 studies), including one study from North America (WMD 12.70 [11.64–13.77]). Most of the underlying comorbidities remained significant risk factors for death except for hepatic disease (pooled RR 1.99 [0.995–3.98], I^2^ = 52.0%, 5 studies). The pooled RRs for the comorbidities were: hypertension, 2.76 [1.95–3.90], I^2^ = 65.1%, 11 studies; cerebral disease, 2.72 [1.41–5.26], I^2^ = 87.2%, 8 studies; cardiac disease, 2.45 [1.97–3.05], I^2^ = 82.3%, 13 studies; respiratory disease, 2.11 [1.52–2.92], I^2^ = 56.1%, 13 studies; malignancy, 1.75 [1.37–2.23], I^2^ = 70.4%, 12 studies; renal disease, 1.62 [1.55–1.70], I^2^ = 0.0%, 12 studies; and diabetes, 1.42 [1.21–1.67], I^2^ = 58.8%, 12 studies. Symptoms of dyspnea (pooled RR 2.86 [1.35–6.07], I^2^ = 82.9%, 8 studies), anorexia (pooled RR 2.16 [1.02–4.61], I^2^ = 5.8%, 3 studies), and fatigue (pooled RR 1.62 [1.05–2.51], I^2^ = 0.0%, 5 studies) were associated with higher risk of death. The extent of heterogeneity in these categorical variables was largely reduced when further analyses were performed according to each continent. The association between laboratory findings and death was largely consistent with the main analyses, except for troponin (pooled SMD 1.24 [− 0.56 to 3.03], I^2^ = 98.6%, 3 studies), prothrombin time (pooled SMD 0.23 [− 0.16 to 0.61], I^2^ = 69.7%, 4 studies), and hemoglobin (pooled SMD − 0.11 [− 0.35 to 0.14], I^2^ = 63.0%, 7 studies). The results of the sensitivity analysis are summarized in Supplementary Fig. S5, and the details are described in Supplementary Table S4.

## Discussion

This is the first study to summarize the risk factors for the critical outcomes (death, admission to the ICU, or critical type of COVID-19) of COVID-19 according to the location of infected patients. It is also the largest updated systematic review regarding risk factors for the poor prognosis of patients with COVID-19. While most risk factors were largely similar across the three continents, several differences were noted. The presence of respiratory disease was associated with a higher risk of the critical outcome in Asia and Europe, but not North America. The presence of hepatic disease was associated with a higher risk of the critical outcome in Europe, but not in Asia and North America. Symptoms of vomiting, anorexia, dizziness, and fatigue were significantly associated with the critical outcome in Asia, but not Europe and North America. While platelet count was inversely associated with the critical outcome in Asia, it was not in Europe and North America. In contrast, lower hemoglobin levels were associated with the poor outcome in Europe but not in Asia and North America.

Our findings of the overall population are consistent with those of previous reviews. Male sex, older age, underlying comorbidities, and several laboratory parameters have been repeatedly emphasized as risk factors for poor outcomes in patients with COVID-19^9,10,99–103^. This disease is well-known for male-sex predominant deterioration. A nationwide study from Denmark reported that male sex was an independent risk factor for death even after adjusting for age and comorbidities^104^. The underlying mechanism for this observation has not yet been elucidated but may be explained by the immune regulatory genes encoded by the X chromosome, which makes men more susceptible to viral infections as compared to women^105^. In addition, sex hormones may act directly in innate immune cells to regulate their function, and indirectly via non-immune cells resulting in immune cell actions^106^. Older age was also a risk factor for grave prognosis among COVID-19 patients in previous systematic reviews^9,10^. This is easily understandable as old age is also a well-known risk factor for death among patients with community-acquired pneumonia and influenza^107–109^. Among many symptoms, dyspnea was the only symptom that was consistently associated with a higher risk of the critical outcome in all three continents, a finding concordant with those in previous reviews^9,102^. Dyspnea is relatively uncommon among COVID-19 patients despite typical lung involvement^2,110^. Therefore, the presence of dyspnea could imply extensive involvement of the lung and lead to poor outcomes.

The results of our study not only confirm previous knowledge regarding the risk factors for the deterioration of COVID-19 patients but also reveal some novel findings. First, some risk factors revealed inter-continental differences. Recognizing such differences can aid the development of proper guidelines for the management of patients according to their region and ethnicity. As noted, underlying respiratory disease was associated with the critical outcomes in Asia and Europe, but not in North America. Although the exact reason for this disparity is beyond the scope of our review, it may be partly explained by the differences in therapies for the treatment of these chronic respiratory diseases^111^. In China, only about 56% of patients with chronic obstructive pulmonary disease receive treatments that are standard in Western countries, while 23% receive Chinese traditional treatments^111^. Considering the protective effect of corticosteroids in the treatment of COVID-19^112^, such a gap in the treatment of chronic respiratory disease may have led to different outcomes among continents. Underlying hepatic disease also showed different impacts on critical outcomes across the three continents. This may be partly due to differences in per capita alcohol consumption, which is higher in Europe compared to North America and Asia^113^. Alcohol consumption is also associated with mortality rates among patients with liver cirrhosis^114^ and also increases the severity of respiratory viral infection and pneumonia^115,116^. Thus, patients from Europe with hepatic disease may have had worse prognoses compared to those in patients from North America and Asia. Among the symptoms of COVID-19, vomiting, anorexia, dizziness, and fatigue were risk factors in Asia, but not in Europe and North America. These symptoms can be associated with weight loss and poor nutritional status during the course of the disease, while BMI is mostly higher for individuals living in Europe and North America, compared to those in Eastern Asian countries^117^. Although our meta-analysis suggested that Hispanic patients may have better prognosis compared to non-Hispanic white, the impact of ethnicity on the prognosis of COVID-19 is yet to be explained. While a regional study from the United States reported that ethnicity may be a factor for diverse outcomes^118^, other studies denied these findings after adjusting for risk factors ^119,120^. A recent meta-analysis of has suggested that, after adjusting patient characteristics, ethnicity may not be an independent prognostic factor^121^.

Second, with enough pooled analysis, various comorbidities are proven to be risk factors. Because viral infections can cause a systemic inflammatory response which can induce myocardial injury and vascular inflammation^122,123^, studies have focused on diseases associated with cardiovascular outcomes as risk factors. In previous systematic reviews including 13, 16, 25, and 36 studies^9,99,101,124^, underlying cardiovascular disease, hypertension, diabetes, congestive heart failure, cerebrovascular disease, chronic kidney disease, respiratory disease, and cancer were identified as risk factors for poor patient outcome. The reviews did not find or mention any significant impact of underlying liver disease. However, several studies have inferred the impact of liver disease on the prognosis of COVID-19. For example, laboratory abnormalities associated with hepatic dysfunction were frequently observed in patients with COVID-19, and were more common in severe forms of COVID-19^125^. Furthermore, a pooled analysis showed a higher incidence of acute hepatic injury in severe COVID-19 compared to that in non-severe disease^126^.

To correctly acknowledge our study findings, several limitations should be noted. First, most of the included studies had retrospective design. This was inevitable because COVID-19 is a novel disease that caused a sudden pandemic. Second, residual heterogeneity was observed in the analyses of continuous variables. The residual extent of heterogeneity may be partially explained by differences in the reported forms of the variables (i.e., mean and SD, median and range, median, and interquartile range), age distribution, level of care, medication details, and nutritional status among studies. Third, some key factors, such as pregnancy, could not be evaluated because they were not commonly reported^127^.

Our extensive systematic review summarized the risk factors associated with the critical outcome (death, admission to the ICU, and critical type of COVID-19) of COVID-19 patients according to location of infected patients (Asia, Europe, and North America). Although the risk factors were mostly consistent across the three continents, underlying diseases, patient symptoms, and laboratory findings posed different impact on patient prognosis in each location. Future studies are required to understand the reasons for such discrepancy.

## Supplementary Information

Supplementary Information 1.

Supplementary Information 2.

Supplementary Information 3.

Supplementary Figures and Tables.

## Data Availability

The data used in this systematic review is available from the corresponding author with a reasonable request.
