# Supplementary material for: A systematic review and meta-analysis of regional risk factors for critical outcomes of COVID-19 during early phase of the pandemic
Source: Sci Rep. 2021 May 7;11:9784. doi: 10.1038/s41598-021-89182-8 (PMC8105319; doi:10.1038/s41598-021-89182-8)
Supplement: Supplementary file 1 — Supplementary Information 1. [file 41598_2021_89182_MOESM1_ESM.docx]

**Title: A systematic review and meta-analysis of regional risk factors for critical outcomes of COVID-19 during early phase of the pandemic**

**Authors: Hyung-Jun Kim, Hyeontaek Hwang, Hyunsook Hong, Jae-Joon Yim, and Jinwoo Lee**

**Detailed search strategy for the systematic review**

**PubMed**

#1. COVID-19

(“COVID-19”[Supplementary Concept] OR “severe acute respiratory syndrome coronavirus 2”[Supplementary Concept] OR COVID19[tiab] OR “COVID 19″[tiab] OR COVID-19[tiab] OR 2019-nCoV[tiab] OR 2019nCoV[tiab] OR nCoV[tiab] OR “novel coronavirus”[tiab] OR “novel CoV”[tiab]) OR ((Wuhan[tiab] OR Hubei[tiab] OR Huanan[tiab]) AND (coronavirus[tiab] OR coronaviruses[tiab] OR betacoronavirus[tiab] OR CoV[tiab] OR nCoV[tiab] OR “coronavirus”[mesh] OR “betacoronavirus”[mesh] OR “coronavirus infections”[mesh] OR pneumonia[tiab] OR outbreak[tiab]))

#2. Hospitalized patients

"hospitalization"[MeSH] OR Hospital*[tiab] OR Admission*[tiab] OR Admit*[tiab]

#3. Outcomes

Critical*[tiab] OR Intensive[tiab] OR ICU[tiab] OR “Critical illness”[Mesh] OR “Critical care”[Mesh] OR “Intensive care units”[Mesh] OR Mortality[tiab] OR Death[tiab] OR Surviv*[tiab] OR Fatal*[tiab] OR “Mortality”[Mesh] OR “Death”[Mesh] OR “Survival”[Mesh] OR Composite[tiab] OR “Risk factors”[Mesh] OR (risk factor*)[tiab]

#4. Children

"child"[MeSH] OR child*[title]

(#1 AND #2 AND #3) NOT #4

**EMBASE**

#1. COVID-19

(COVID19:ab,ti OR ‘COVID 19’:ab,ti OR COVID-19:ab,ti OR 2019-nCoV:ab,ti OR 2019nCoV:ab,ti OR nCoV:ab,ti OR ‘novel coronavirus’:ab,ti OR ‘novel CoV’:ab,ti) OR ((Wuhan:ab,ti OR Hubei:ab,ti OR Huanan:ab,ti) AND (coronavirus:ab,ti OR coronaviruses:ab,ti OR betacoronavirus:ab,ti OR CoV:ab,ti OR nCoV:ab,ti OR ‘Coronavirinae’/exp OR ‘betacoronavirus’/exp OR ‘coronavirus infection’/exp OR pneumonia:ab,ti OR outbreak:ab,ti))

#2. Hospitalized patients

‘hospitalization’/exp OR ‘hospital admission’/exp OR ‘hospital readmission’/exp OR Hospital*:ab,ti OR Admission*:ab,ti OR Admit*:ab,ti

#3. Outcomes

Critical*:ab,ti OR Intensive:ab,ti OR ICU:ab,ti OR ‘critical illness’/exp OR ‘intensive care’/exp OR ‘intensive care unit’/exp OR Mortality:ab,ti OR Death:ab,ti OR Surviv*:ab,ti OR Fatal*:ab,ti OR ‘mortality’/exp OR ‘Death’/exp OR ‘survival’/exp OR Composite:ab,ti OR ‘composite outcome’/exp OR ‘risk factor’/exp OR (risk factor*):ab,ti

#4. Children

‘child’/exp OR child*:ti

(#1 AND #2 AND #3) NOT #4

**Cochrane library**

#1. COVID-19

((Wuhan:ti,ab,kw OR Hubei:ti,ab,kw OR Huanan:ti,ab,kw) AND ([mh coronavirus] OR [mh betacoronavirus] OR [mh “coronavirus infections”] OR coronavirus:ti,ab,kw OR coronaviruses:ti,ab,kw OR betacoronavirus:ti,ab,kw OR CoV:ti,ab,kw OR nCoV:ti,ab,kw OR pneumonia:ti,ab,kw OR outbreak:ti,ab,kw)) OR (COVID19:ti,ab,kw OR (COVID 19):ti,ab,kw OR COVID-19:ti,ab,kw OR nCoV:ti,ab,kw OR “novel coronavirus”:ti,ab,kw)

#2. Hospitalized patients

[mh hospitalization] OR Hospital*:ti,ab,kw OR Admission*:ti,ab,kw OR Admit*:ti,ab,kw

#3. Outcomes

[mh “Critical illness”] OR [mh “Intensive care units”] OR [mh Mortality] OR [mh Death] OR [mh Survival] OR [mh “Risk factors”] OR [mh “Critical care”] OR Critical*:ti,ab,kw OR Intensive:ti,ab,kw OR ICU:ti,ab,kw OR Mortality:ti,ab,kw OR Death:ti,ab,kw OR Surviv*:ti,ab,kw OR Fatal*:ti,ab,kw OR Composite:ti,ab,kw OR (risk factor*):ti,ab,kw

#4. Children

[mh child] OR child*:ti

(#1 AND #2 AND #3) NOT #4

**Web of science**

#1. COVID-19

TS = ((COVID19 OR “COVID 19” OR COVID-19 OR 2019-nCoV OR 2019nCoV OR nCoV OR “novel coronavirus” OR “novel CoV”) OR ((Wuhan OR Hubei OR Huanan) AND (coronavirus* OR betacoronavirus* OR CoV OR nCoV OR pneumonia OR outbreak)))

#2. Hospitalized patients

TS = (Hospital* OR Admission* OR Admit*)

#3. Outcomes

TS = (Critical* OR Intensive OR ICU OR Mortality OR Death OR Surviv* OR Fatal* OR Composite OR (risk factor*))

#4. Children

TS = child*

(#1 AND #2 AND #3) NOT #4
