# Supplementary material for: A systematic review and meta-analysis of regional risk factors for critical outcomes of COVID-19 during early phase of the pandemic
Source: Sci Rep. 2021 May 7;11:9784. doi: 10.1038/s41598-021-89182-8 (PMC8105319; doi:10.1038/s41598-021-89182-8)
Supplement: Supplementary file 4 — Supplementary Figures and Tables. [file 41598_2021_89182_MOESM4_ESM.docx]

**Title: A systematic review and meta-analysis of regional risk factors for critical outcomes of COVID-19 during early phase of the pandemic**

**Authors: Hyung-Jun Kim, Hyeontaek Hwang, Hyunsook Hong, Jae-Joon Yim, and Jinwoo Lee**

**Supplementary Fig. S1** Flow diagram of the systematic literature review


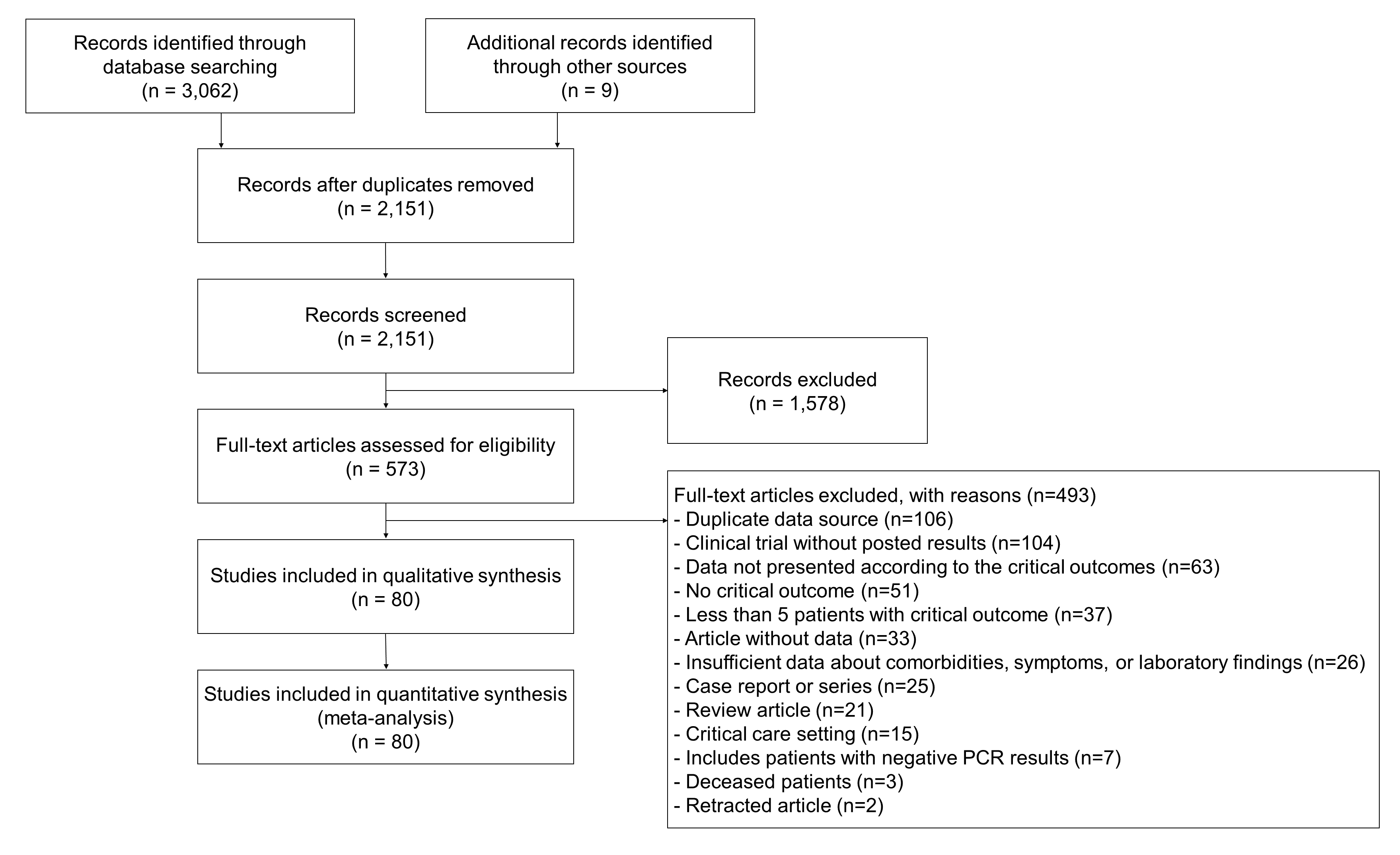


**Supplementary Fig. S2** Impact of male sex on composite outcome including death, intensive care unit admission, and critical type of COVID-19.


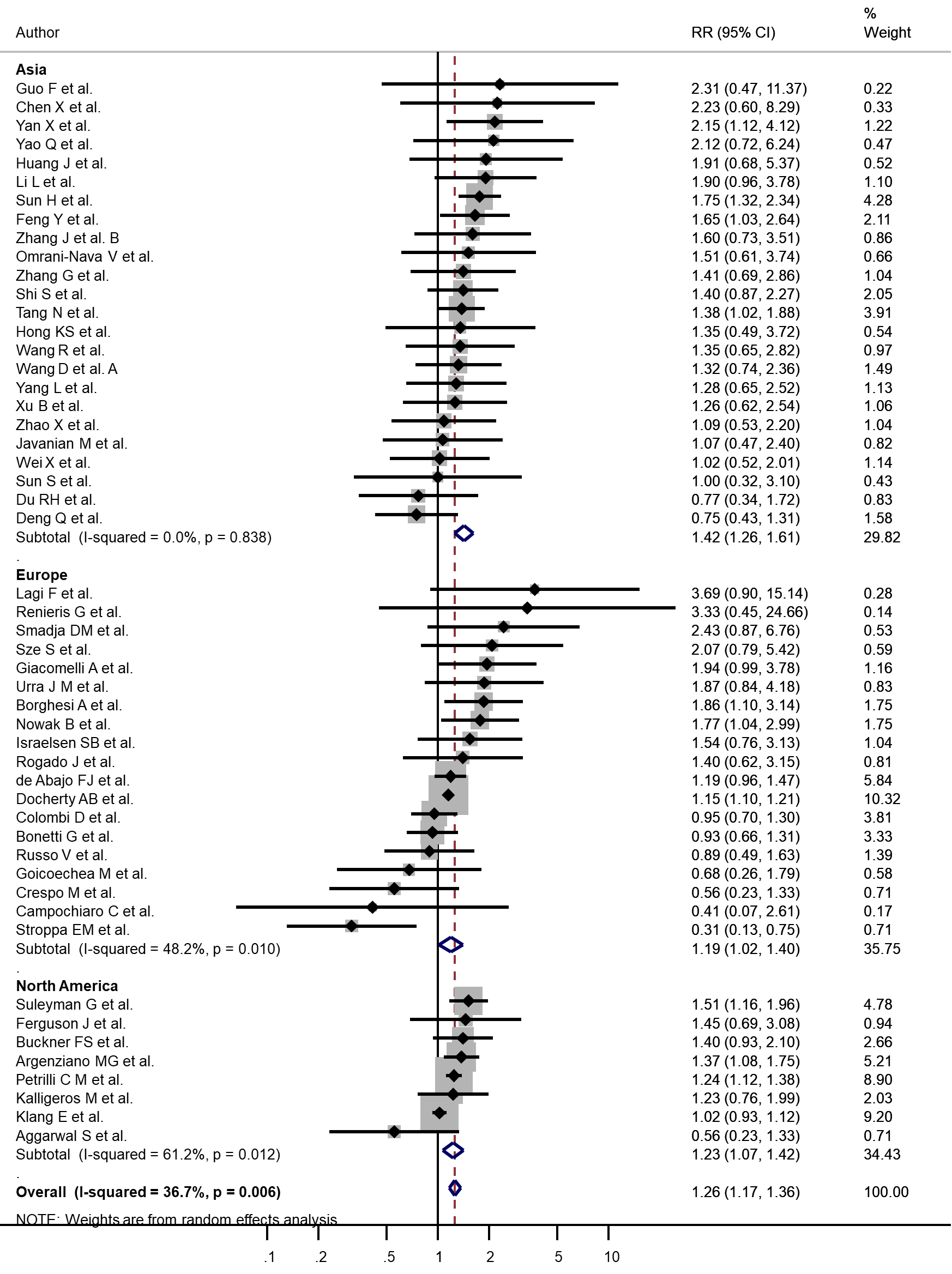


Abbreviation: RR, relative risk

**Supplementary Fig. S3** Impact of patient age on the critical outcome including death, intensive care unit admission, and critical type of COVID-19.


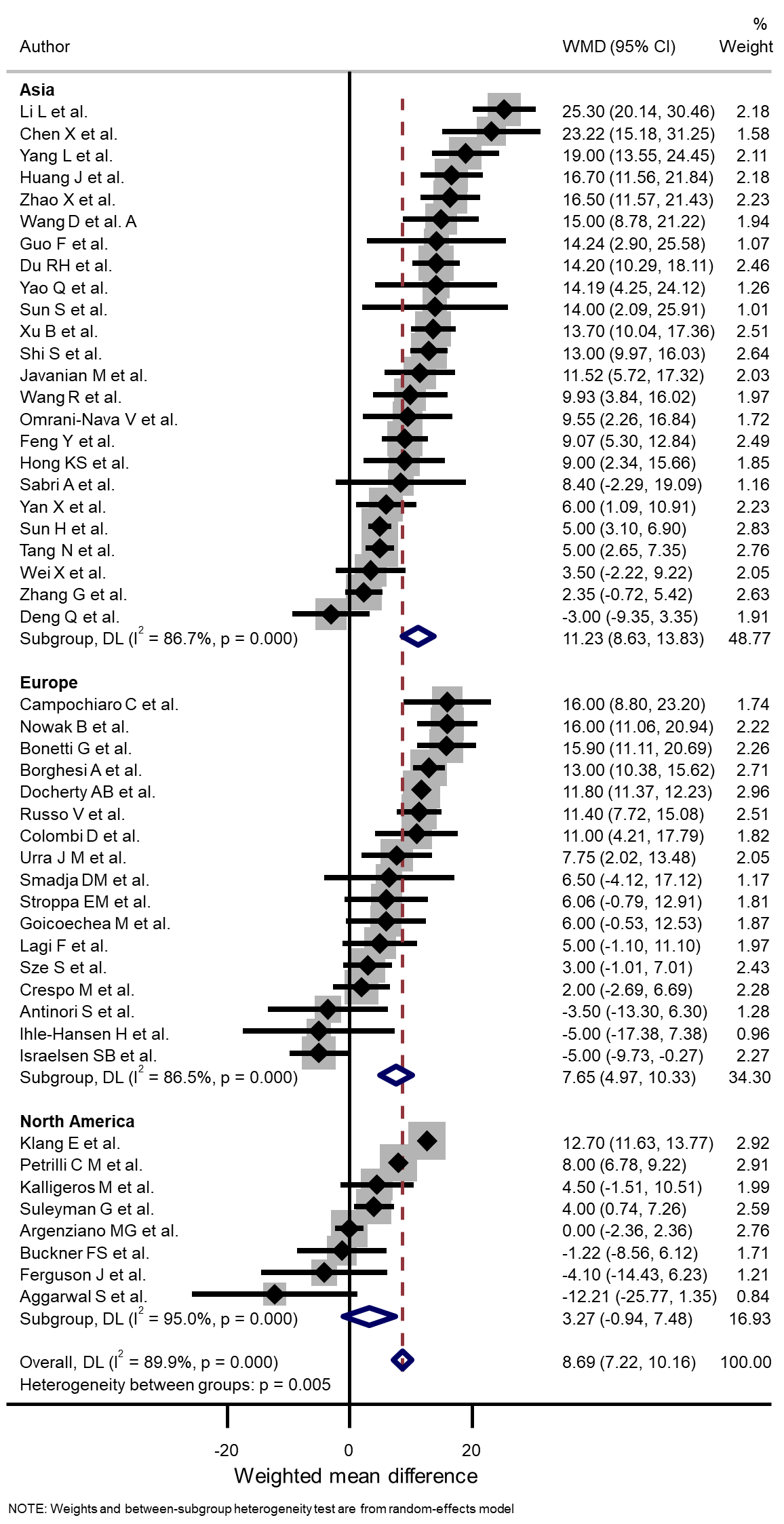


Abbreviation: WMD, weighted mean difference

**Supplementary Fig. S4** Association between ethnicity and the critical outcome including intensive care unit admission and death compared to non-Hispanic white ethnicity with a random effects model. a) Hispanic ethnicity. b) non-Hispanic black ethnicity. c) Asian ethnicity.


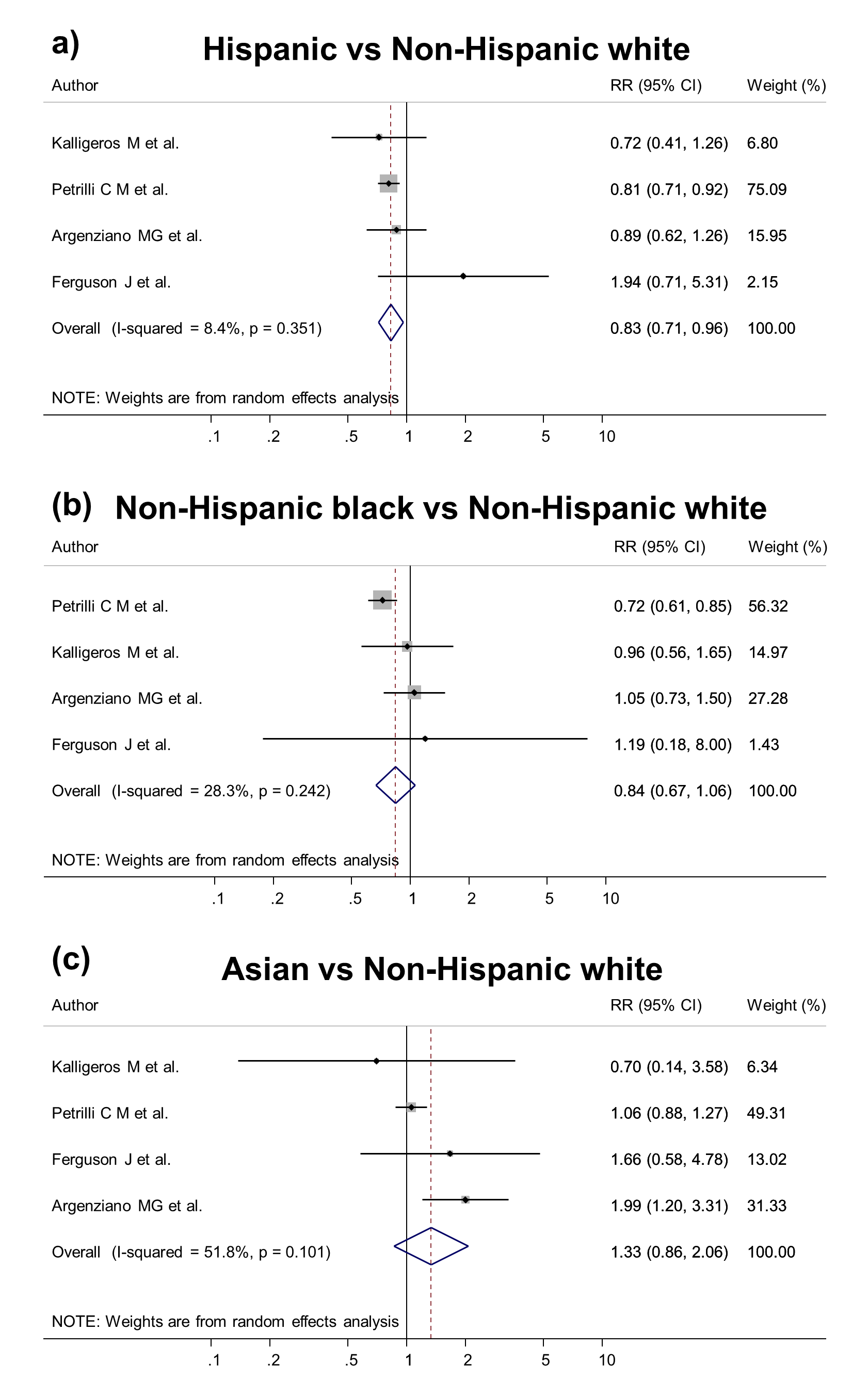


**Supplementary Figure S5.** Impact of (a) underlying condition, (b) patient symptom, and (c) laboratory findings on death of patients with COVID-19.

The number in the parenthesis represents the number of studies included in the meta-analysis.

Abbreviations: LDH, lactate dehydrogenase; BUN, blood urea nitrogen; AST, aspartate transaminase; CK, creatine kinase; WBC, white blood cell; ALT, alanine transaminase; PT, prothrombin time.

**Supplementary Table S1.** Definition of underlying disease mentioned in each study

| Study | Cardiac disease | Renal disease | Malignancy | Respiratory disease | Hepatic disease | Cerebral disease |
| --- | --- | --- | --- | --- | --- | --- |
| Aggarwal S et al. | Coronary artery disease | Chronic kidney disease | Malignancy | COPD | – | Cerebrovascular disease |
| Antinori S et al. | – | – | Cancer | – | – | – |
| Argenziano MG et al. | Coronary artery disease | Renal disease | Active cancer | Pulmonary disease | Cirrhosis | History of stroke |
| Bonetti G et al. | Cardiovascular disease | Chronic kidney diseases | Cancer | Chronic respiratory diseases | – | – |
| Borghesi A et al. | Cardiovascular disease | – | Oncological history within the past 5 years | Chronic obstructive/restrictive lung disease | – | – |
| Buckner FS et al. | Cardiovascular disease | Chronic kidney disease | Cancer | COPD | – | – |
| Campochiaro C et al. | Coronary artery disease | Chronic kidney disease | Cancer | COPD | – | – |
| Chen C et al. | Past coronary heart disease history | – | – | – | – | – |
| Chen X et al. | Heart disease | – | Malignancy | Pulmonary disease | Hepatic disease | Cerebral disease |
| Colombi D et al. | Cardiovascular disease | Chronic kidney failure | Oncological disease | Pulmonary disease | Hepatic failure | Neurological disease |
| Crespo M et al. | Heart disease | End-stage renal disease with kidney transplant | Cancer | Lung disease | – | – |
| de Abajo FJ et al. | Ischemic heart disease | Chronic renal failure | Malignancy | COPD | – | Cerebrovascular disease |
| Deng Q et al. | Coronary heart disease | – | – | COPD | – | – |
| Docherty AB et al. | Chronic cardiac disease | Chronic kidney disease | Malignancy | Chronic pulmonary disease | Moderate/severe liver disease | Chronic neurological disorder |
| Du RH et al. | – | – | Malignancy | – | – | – |
| Feng Y et al. | Not mentioned | Chronic nephropathy | Malignancy | COPD | – | Cerebrovascular disease |
| Ferguson J et al. | Coronary artery disease | Chronic kidney disease | – | Pulmonary disease | – | Cerebrovascular disease |
| Giacomelli A et al. | – | – | – | – | – | – |
| Goicoechea M et al. | Coronary heart disease | – | – | COPD | – | – |
| Guo F et al. | – | – | – | – | – | – |
| He W et al. | – | – | – | – | – | – |
| Hong KS et al. | Cardiovascular disease | – | Malignancy | Chronic lung disease | Liver disease | Cerebrovascular disease |
| Huang C et al. | Cardiovascular disease | – | Malignancy | COPD | Chronic liver disease | – |
| Huang J et al. | Coronary heart disease | – | Cancer | COPD | – | Cerebrovascular disease |
| Huang JT et al. | – | – | – | – | – | – |
| Ihle-Hansen H et al. | – | – | – | – | – | – |
| Israelsen SB et al. | Cardiovascular disease | – | – | COPD | – | – |
| Javanian M et al. | Cardiovascular disease | Chronic kidney disease | Malignancy | COPD | Liver disease | Cerebrovascular disease |
| Ji M et al. | – | – | – | – | – | – |
| Kalligeros M et al. | Heart disease | Chronic renal disease | Cancer | Lung disease | Cirrhosis | – |
| Klang E et al. | Coronary artery disease | Chronic kidney disease | Cancer | – | – | – |
| Lagi F et al. | Coronary heart disease | Chronic renal failure | Former or still oncologic diseases | COPD | Chronic hepatitis B | Cerebrovascular disease |
| Lee LYW et al. | Cardiovascular disease | – | – | COPD | – | – |
| Li J et al. A | Coronary heart disease | Chronic renal disease | Solid tumor | Pulmonary disease | – | Neurological disease |
| Li J et al. B | – | – | – | – | – | – |
| Li L et al. | Cardiovascular disease | – | Malignancy | COPD | – | – |
| Luo X et al. | – | – | – | – | – | – |
| Lv Z et al. | Coronary heart disease | – | Carcinoma | COPD | – | – |
| Nowak B et al. | Cardiovascular disease | Chronic renal disease | Malignancy | COPD | – | – |
| Omrani-Nava V et al. | Cardiac disease | – | Malignancy | Asthma | – | – |
| Pan F et al. | Cardiovascular disease | – | – | COPD | – | – |
| Pan L et al. | – | – | Malignant tumor | Respiratory system disease | – | Nervous system disease |
| Pei G et al. | – | – | – | – | – | – |
| Peng Y et al. | – | – | – | – | – | – |
| Pereira MR et al. | – | Chronic kidney disease | Active cancer | Chronic lung disease | – | – |
| Petrilli C M et al. | Coronary artery disease | Chronic kidney disease | Cancer | Asthma | – | – |
| Renieris G et al. | Coronary heart disease | Chronic renal disease | – | COPD | – | – |
| Rogado J et al. | Cardiovascular disease | Chronic kidney disease | Cancer | COPD | – | – |
| Russo V et al. | Heart failure | Chronic kidney disease | – | COPD | – | Previous ischemic stroke |
| Sabri A et al. | Ischemic heart disease | – | – | – | – | – |
| Shi Q et al. | Cardiovascular disease | Chronic kidney disease | Malignancy | Chronic pulmonary disease | Chronic liver disease | Cerebrovascular disease |
| Shi S et al. | Coronary heart disease | Chronic renal disease | Cancer | COPD | – | Cerebrovascular disease |
| Smadja DM et al. | Coronary heart disease | Chronic kidney disease | Cancer | – | – | Stroke |
| Stroppa EM et al. | – | – | Malignancy | Chronic obstructive pulmonary disease | – | – |
| Suleyman G et al. | Coronary artery disease | Chronic kidney disease | Cancer | COPD | – | – |
| Sun H et al. | Coronary heart disease | – | – | Previous respiratory disease | – | Consciousness disorders |
| Sun S et al. | – | – | – | – | – | – |
| Sze S et al. | Ischemic heart disease | Chronic kidney disease | Cancer | COPD | – | Stroke |
| Tambe M P et al. | Cardiovascular disease | Chronic kidney disease | – | COPD | Alcoholic liver disease | – |
| Tang N et al. | – | – | – | – | – | – |
| Urra J M et al. | Cardiovascular disease | – | Cancer | COPD | – | – |
| Valeri A M et al. | Coronary artery disease | Chronic kidney disease | – | Pulmonary disease | – | – |
| Wang D et al. A | Cardiovascular disease | Chronic kidney disease | Malignancy | COPD | Chronic liver disease | Cerebrovascular disease |
| Wang D et al. B | Cardiovascular disease | Chronic kidney disease | – | COPD | Chronic liver disease | Cerebrovascular disease |
| Wang F et al. | Cardiovascular disease | Chronic kidney disease | – | Chronic pulmonary disease | Chronic liver disease | Cerebrovascular disease |
| Wang K et al. | Coronary heart disease | Chronic kidney disease | Cancer | COPD | – | Cerebrovascular disease |
| Wang R et al. | – | – | – | – | – | – |
| Wei X et al. | Cardiovascular disease | – | – | – | – | – |
| Xu B et al. | – | Kidney disease | – | Lung disease | – | – |
| Yan X et al. | CHD (abbreviation not defined) | Kidney diseases other than acute kidney injury | Tumor | COPD | Liver cirrhosis | Cerebrovascular disease |
| Yang L et al. | Chronic heart disease | Chronic kidney disease | Malignancy | Chronic lung disease | Chronic liver disease | – |
| Yao Q et al. | Any patient with hyperlipidemia, coronary heart disease, hemorrhagic stroke, or ischemic stroke | – | Cancer | Any patient with bronchiectasis, COPD, or asthma | Chronic liver disease | – |
| Yuan M et al. | Cardiac disease | – | Tumor | – | – | Cerebral infarction |
| Zhang F et al. | – | – | – | – | – | – |
| Zhang G et al. | – | – | – | – | – | – |
| Zhang J et al. A | Cardiovascular disease | – | Malignancy | COPD | Chronic liver disease | – |
| Zhang J et al. B | Cardiovascular disease | Urinary system disease | Malignant tumor | Pulmonary disease | – | – |
| Zhang J et al. C | Cardiovascular disease | Urinary system disease | Malignant tumor | Respiratory system disease | – | Cerebrovascular disease |
| Zhang X et al. | – | – | – | – | – | – |
| Zhao X et al. | – | – | – | – | – | – |

**Supplementary Table S2.** Evaluation of the quality of studies included in the systematic review

| Study | Study participation | Study attrition | Prognostic factor measurement | Outcome measurement | Confounding measurement and account | Analysis |
| --- | --- | --- | --- | --- | --- | --- |
| Aggarwal S et al. | Partly | Partly | Yes | Yes | Partly | Yes |
| Antinori S et al. | Partly | Yes | Yes | Yes | Partly | Yes |
| Argenziano MG et al. | Yes | Partly | Yes | Yes | Yes | Yes |
| Bonetti G et al. | Yes | Yes | Yes | Yes | Partly | Yes |
| Borghesi A et al. | Partly | Yes | Partly | Yes | Partly | Yes |
| Buckner FS et al. | Partly | Partly | Yes | Yes | Partly | Yes |
| Campochiaro C et al. | Partly | Yes | Partly | Yes | Partly | Yes |
| Chen C et al. | Yes | Partly | Partly | Yes | Partly | Yes |
| Chen X et al. | Yes | Partly | Partly | Yes | Partly | Yes |
| Colombi D et al. | Partly | Partly | Partly | Yes | Partly | Yes |
| Crespo M et al. | Partly | Partly | Partly | Yes | Partly | Yes |
| de Abajo FJ et al. | Yes | Partly | No | Yes | No | Yes |
| Deng Q et al. | Yes | Partly | Partly | Yes | Partly | Yes |
| Docherty AB et al. | Yes | Yes | Partly | Yes | Partly | Yes |
| Du RH et al. | Yes | Partly | Yes | Yes | Partly | Yes |
| Feng Y et al. | Yes | Partly | Yes | Yes | Yes | Yes |
| Ferguson J et al. | Yes | Partly | Yes | Yes | Yes | Yes |
| Giacomelli A et al. | Yes | Partly | Yes | Yes | Partly | Yes |
| Goicoechea M et al. | Partly | Partly | Partly | Yes | Partly | Yes |
| Guo F et al. | Partly | Partly | Partly | Yes | Partly | Yes |
| He W et al. | Partly | Partly | Partly | Yes | Partly | Yes |
| Hong KS et al. | Partly | Partly | Yes | Yes | Partly | Yes |
| Huang C et al. | Yes | Partly | Yes | Yes | Yes | Yes |
| Huang J et al. | Yes | Partly | Partly | Yes | Partly | Yes |
| Huang JT et al. | Yes | Yes | Yes | Yes | Partly | Yes |
| Ihle-Hansen H et al. | Partly | Partly | Partly | Yes | Partly | Yes |
| Israelsen SB et al. | Partly | Partly | Yes | Yes | Partly | Yes |
| Javanian M et al. | Partly | Partly | Yes | Yes | Partly | Yes |
| Ji M et al. | Yes | Yes | Partly | Yes | No | Yes |
| Kalligeros M et al. | Partly | Partly | Partly | Yes | Partly | Yes |
| Klang E et al. | Yes | Yes | Partly | Yes | Partly | Yes |
| Lagi F et al. | Partly | Partly | Partly | Yes | Partly | Yes |
| Lee LYW et al. | Partly | Partly | Yes | Yes | Partly | Yes |
| Li J et al. A | Partly | Partly | Yes | Yes | Partly | Yes |
| Li J et al. B | Partly | Partly | Partly | Yes | Partly | Yes |
| Li L et al. | Partly | Partly | Yes | Yes | Yes | Yes |
| Luo X et al. | Yes | Partly | Yes | Yes | Yes | Yes |
| Lv Z et al. | Partly | Yes | Partly | Yes | Partly | Yes |
| Nowak B et al. | Partly | Partly | Partly | Yes | Partly | Yes |
| Omrani-Nava V et al. | Partly | Partly | No | Yes | No | Yes |
| Pan F et al. | Yes | Yes | Yes | Yes | Yes | Yes |
| Pan L et al. | Partly | Partly | Yes | Yes | Partly | Yes |
| Pei G et al. | Yes | Partly | Partly | Yes | Partly | Yes |
| Peng Y et al. | No | Partly | Partly | Yes | Partly | Yes |
| Pereira MR et al. | Partly | Partly | Partly | Yes | Partly | Yes |
| Petrilli C M et al. | Yes | Yes | Partly | Yes | Partly | Yes |
| Renieris G et al. | Partly | Partly | Partly | Yes | No | Yes |
| Rogado J et al. | Partly | Partly | Partly | Yes | Partly | Yes |
| Russo V et al. | Yes | Partly | Partly | Yes | Partly | Yes |
| Sabri A et al. | Yes | Partly | Partly | Yes | No | Yes |
| Shi Q et al. | Partly | Partly | Yes | Yes | Yes | Yes |
| Shi S et al. | Yes | Partly | Yes | Yes | Partly | Yes |
| Smadja DM et al. | Partly | Partly | Yes | Yes | Partly | Yes |
| Stroppa EM et al. | Partly | Partly | Partly | Yes | Partly | Yes |
| Suleyman G et al. | Yes | Partly | Yes | Yes | Yes | Yes |
| Sun H et al. | Partly | Partly | Partly | Yes | Partly | Yes |
| Sun S et al. | Partly | Partly | Partly | Yes | Partly | Yes |
| Sze S et al. | Yes | Partly | Partly | Yes | Partly | Yes |
| Tambe M P et al. | Yes | Partly | Partly | Yes | No | Yes |
| Tang N et al. | Yes | Partly | Partly | Yes | No | Yes |
| Urra J M et al. | Partly | Partly | Partly | Yes | Partly | Yes |
| Valeri A M et al. | Partly | Partly | Partly | Yes | Partly | Yes |
| Wang D et al. A | Yes | Partly | Yes | Yes | Yes | Yes |
| Wang D et al. B | Yes | Partly | Yes | Yes | Yes | Yes |
| Wang F et al. | No | Partly | Yes | Yes | Partly | Yes |
| Wang K et al. | Yes | Partly | Yes | Yes | Partly | Yes |
| Wang R et al. | Yes | Partly | Partly | Yes | Partly | Yes |
| Wei X et al. | Yes | Partly | Yes | Yes | Partly | Yes |
| Xu B et al. | Yes | Partly | Partly | Yes | Partly | Yes |
| Yan X et al. | Yes | Yes | Partly | Yes | Partly | Yes |
| Yang L et al. | Yes | Partly | Partly | Yes | Partly | Yes |
| Yao Q et al. | Yes | Partly | Partly | Yes | Partly | Yes |
| Yuan M et al. | Partly | Yes | Partly | Yes | Partly | Partly |
| Zhang F et al. | Partly | Partly | Partly | Yes | Partly | Yes |
| Zhang G et al. | Partly | Partly | Partly | Yes | Partly | Yes |
| Zhang J et al. A | Yes | Partly | Partly | Yes | Partly | Yes |
| Zhang J et al. B | Yes | Partly | Partly | Yes | Partly | Yes |
| Zhang J et al. C | Yes | Yes | Partly | Yes | Partly | Yes |
| Zhang X et al. | Yes | Yes | Partly | Yes | No | Yes |
| Zhao X et al. | Yes | Partly | Partly | Yes | No | Yes |

**Supplementary Table S3**. Degree of heterogeneity and publication bias according to each analysis

| Variables | | Number of studies included in analysis | I^2^ for heterogeneity | *P* for heterogeneity | *P* for Egger's test |
| --- | --- | --- | --- | --- | --- |
| Male sex | | 51 | 36.7% | 0.006 | 0.042 |
| Age | | 50 | 83.5% | <0.001 | 0.373 |
| Body mass index | | 8 | 77.1% | <0.001 | 0.114 |
| Underlying condition | |  |  |  |  |
|  | Diabetes | 46 | 58.0% | <0.001 | 0.001 |
|  | Hypertension | 45 | 71.2% | <0.001 | 0.432 |
|  | Cardiac disease | 45 | 74.7% | <0.001 | 0.324 |
|  | Respiratory disease | 44 | 64.9% | <0.001 | 0.755 |
|  | Malignancy | 36 | 51.6% | <0.001 | 0.020 |
|  | Renal disease | 31 | 78.0% | <0.001 | 0.542 |
|  | Smoking | 23 | 0.1% | 0.458 | 0.825 |
|  | Cerebral disease | 22 | 82.8% | <0.001 | 0.042 |
|  | Hepatic disease | 16 | 64.5% | <0.001 | 0.668 |
| Patient symptom | |  |  |  |  |
|  | Fever | 32 | 14.6% | 0.235 | 0.200 |
|  | Cough | 31 | 55.1% | <0.001 | 0.994 |
|  | Dyspnea | 28 | 85.7% | <0.001 | 0.019 |
|  | Diarrhea | 25 | 0.0% | 0.609 | 0.724 |
|  | Myalgia | 22 | 37.2% | 0.042 | 0.630 |
|  | Headache | 19 | 26.6% | 0.139 | 0.844 |
|  | Fatigue | 17 | 62.8% | <0.001 | 0.066 |
|  | Sputum | 16 | 65.1% | <0.001 | 0.808 |
|  | Sore throat | 15 | 45.5% | 0.029 | 0.063 |
|  | Nausea | 12 | 59.7% | 0.004 | 0.682 |
|  | Chest tightness | 10 | 43.0% | 0.072 | 0.568 |
|  | Anorexia | 8 | 69.8% | 0.002 | 0.074 |
|  | Vomiting | 7 | 48.8% | 0.069 | 0.045 |
|  | Rhinorrhea | 5 | 21.0% | 0.281 | 0.441 |
|  | Dizziness | 5 | 5.1% | 0.378 | 0.050 |
|  | Abdominal pain | 5 | 87.0% | <0.001 | 0.554 |
| Laboratory findings | |  |  |  |  |
|  | Lymphocyte count | 37 | 76.8% | <0.001 | 0.003 |
|  | WBC count | 34 | 92.6% | <0.001 | 0.585 |
|  | Creatinine | 32 | 84.3% | <0.001 | 0.546 |
|  | Platelet count | 31 | 72.5% | <0.001 | 0.489 |
|  | ALT | 30 | 30.2% | 0.062 | 0.012 |
|  | Neutrophil count | 28 | 92.7% | <0.001 | 0.859 |
|  | Hemoglobin | 26 | 67.8% | <0.001 | 0.215 |
|  | AST | 26 | 87.4% | <0.001 | 0.023 |
|  | D-dimer | 25 | 92.2% | <0.001 | 0.414 |
|  | LDH | 23 | 91.0% | <0.001 | 0.097 |
|  | Total bilirubin | 19 | 54.8% | 0.002 | 0.183 |
|  | CK | 18 | 89.5% | <0.001 | 0.371 |
|  | BUN | 15 | 93.6% | <0.001 | 0.858 |
|  | Troponin^*^ | 14 | 94.7% | <0.001 | 0.747 |
|  | PT | 12 | 32.2% | 0.133 | 0.240 |
|  | Monocyte count | 11 | 79.6% | <0.001 | 0.778 |
|  | proBNP | 5 | 97.6 | <0.001 | 0.653 |

Abbreviations: WBC, white blood cell; ALT, alanine transaminase; AST, aspartate transaminase; LDH, lactate dehydrogenase; CK, creatine kinase; BUN, blood urea nitrogen; PT, prothrombin time; proBNP, *pro* brain-type natriuretic peptide.

^*^Includes both troponin I and troponin T

**Supplementary Table S4.** Detailed results from the sensitivity analysis with selected studies

| Variables | | Asia | n | I^2^ | Europe | n | I^2^ | North America | n | I^2^ | Total | n | I^2^ |
| --- | --- | --- | --- | --- | --- | --- | --- | --- | --- | --- | --- | --- | --- |
| Male sex | | 1.57 (1.12–2.20) | 8 | 22.5% | 1.13 (0.92–1.39) | 6 | 29.8% | 1.02 (0.93–1.13) | 1 | – | 1.17 (1.02–1.34) | 15 | 44.8% |
| Age, WMD | | 13.85 (9.76–17.93) | 8 | 79.6% | 11.23 (7.62–14.8) | 5 | 82.3% | 12.70 (11.64–13.77) | 1 | – | 12.44 (10.76–14.13) | 14 | 79.0% |
| Underlying condition | | |  |  |  |  |  |  |  |  |  |  |  |
|  | Hypertension | 3.50 (2.38–5.12) | 7 | 36.9% | 1.99 (1.06–3.74) | 3 | 21.2% | 1.72 (1.53–1.95) | 1 | – | 2.76 (1.95–3.90) | 11 | 65.1% |
|  | Cerebral disease | 5.07 (3.22–7.98) | 5 | 25.3% | 1.34 (1.25–1.43) | 3 | 0.0% | – | 0 | – | 2.72 (1.41–5.26) | 8 | 87.2% |
|  | Cardiac disease | 4.50 (3.75–6.66) | 7 | 0.0% | 1.56 (1.31–1.86) | 5 | 19.8% | 1.66 (1.51–1.83) | 1 | – | 2.45 (1.97–3.05) | 13 | 82.3% |
|  | Respiratory disease | 3.37 (1.98–5.72) | 8 | 16.8% | 1.58 (1.20–2.08) | 5 | 46.5% | – | 0 | – | 2.11 (1.52–2.92) | 13 | 56.1% |
|  | Hepatic disease | 2.89 (1.26–6.61) | 4 | 18.8% | 1.29 (1.10–1.51) | 1 |  | – | 0 | – | 1.99 (0.995–3.98) | 5 | 52.0% |
|  | Malignancy | 4.58 (2.71–7.74) | 7 | 11.6% | 1.40 (1.31–1.50) | 4 | 0.0% | 1.23 (1.09–1.38) | 1 | – | 1.75 (1.37–2.23) | 12 | 70.4% |
|  | Renal disease | 2.32 (1.47–3.67) | 6 | 0.0% | 1.63 (1.54–1.71) | 5 | 0.0% | 1.58 (1.43–1.75) | 1 | – | 1.62 (1.55–1.70) | 12 | 0.0% |
|  | Diabetes | 2.37 (1.66–3.41) | 6 | 0.0% | 1.16 (1.10–1.23) | 5 | 0.0% | 1.41 (1.28–1.55) | 1 | – | 1.42 (1.21–1.67) | 12 | 58.8% |
|  | Smoking | 1.58 (1.09–2.28) | 4 | 0.0% | 1.24 (1.18–1.31) | 3 | 0.0% | 1.19 (1.07–1.32) | 1 | – | 1.24 (1.18–1.29) | 8 | 0.0% |
| Symptoms | |  |  |  |  |  |  |  |  |  |  |  |  |
|  | Dyspnea | 4.51 (2.37–8.55) | 6 | 59.8% | 0.85 (0.54–1.34) | 2 | 0.0% | – | 0 | – | 2.86 (1.35–6.07) | 8 | 82.9% |
|  | Anorexia | 2.16 (1.02–4.61) | 3 | 5.8% | – | 0 | – | – | 0 | – | 2.16 (1.02–4.61) | 3 | 5.8% |
|  | Vomiting | 1.71 (0.86–3.40) | 3 | 0.0% | – | 0 | – | – | 0 | – | 1.71 (0.86–3.40) | 3 | 0.0% |
|  | Dizziness | 1.67 (0.97–2.88) | 4 | 0.0% | – | 0 | – | – | 0 | – | 1.67 (0.97–2.88) | 4 | 0.0% |
|  | Sputum | 1.63 (0.997–2.67) | 5 | 34.5% | – | 0 | – | – | 0 | – | 1.63 (0.997–2.67) | 5 | 34.5% |
|  | Fatigue | 1.62 (1.05–2.51) | 5 | 0.0% | – | 0 | – | – | 0 | – | 1.62 (1.05–2.51) | 5 | 0.0% |
|  | Nausea | 1.49 (0.52–4.25) | 3 | 28.0% | – | 0 | – | – | 0 | – | 1.49 (0.52–4.25) | 3 | 28.0% |
|  | Chest tightness | 1.46 (0.84–2.56) | 3 | 0.0% | – | 0 | – | – | 0 | – | 1.46 (0.84–2.56) | 3 | 0.0% |
|  | Abdominal pain | 1.42 (0.55–3.65) | 3 | 14.1% | – | 0 | – | – | 0 | – | 1.42 (0.55–3.65) | 3 | 14.1% |
|  | Diarrhea | 1.31 (0.53–3.21) | 6 | 62.0% | 0.18 (0.01–2.44) | 1 | – | – | 0 | – | 1.09 (0.43–2.75) | 7 | 64.3% |
|  | Fever | 0.89 (0.61–1.28) | 8 | 0.0% | 1.39 (0.47–4.08) | 2 | 64.8% | – | 0 | – | 0.95 (0.71–1.28) | 10 | 0.0% |
|  | Headache | 1.10 (0.48–2.50) | 6 | 41.0% | 0.18 (0.01–2.70) | 1 | – | – | 0 | – | 0.94 (0.40–2.21) | 7 | 45.5% |
|  | Myalgia | 0.94 (0.52–1.71) | 5 | 37.6% | 0.39 (0.06–2.60) | 1 | – | – | 0 | – | 0.88 (0.50–1.55) | 6 | 32.5% |
|  | Cough | 0.90 (0.66–1.24) | 8 | 0.0% | 0.71 (0.45–1.10) | 2 | 0.0% | 0.83 (0.64–1.08) | 10 | 0.00% | 0.83 (0.64–1.08) | 10 | 0.0% |
|  | Sore throat | 0.77 (0.35–1.67) | 3 | 0.0% | 0.19 (0.01–2.92) | 1 | – | – | 0 | – | 0.69 (0.32–1.47) | 4 | 2.1% |
| Laboratory findings | | |  |  |  |  |  |  |  |  |  |  |  |
|  | LDH | 2.20 (1.65–2.74) | 4 | 83.6% | 0.92 (0.29–1.55) | 3 | 81.4% | – | 0 | – | 1.62 (0.98–2.26) | 7 | 93.4% |
|  | BUN | 1.68 (0.43–2.94) | 4 | 97.4% | 0.85 (0.13–1.57) | 2 | 54.9% | – | 0 | – | 1.39 (0.46–2.31) | 6 | 96.3% |
|  | Neutrophil count | 1.56 (0.86–2.26) | 8 | 95.6% | 0.60 (0.32–0.89) | 3 | 26.0% | – | 0 | – | 1.29 (0.71–1.87) | 11 | 95.4% |
|  | Troponin^*^ | 1.72 (-0.72–4.16) | 2 | 98.6% | 0.27 (-0.06–0.59) | 1 | – | – | 0 | – | 1.24 (-0.56–3.03) | 3 | 98.6% |
|  | AST | 1.23 (0.73–1.74) | 6 | 88.1% | 0.61 (0.28–0.94) | 1 | – | – | 0 | – | 1.14 (0.67–1.61) | 7 | 89.2% |
|  | WBC count | 1.32 (0.61–2.04) | 9 | 96.2% | 0.39 (0.17–0.61) | 3 | 0.0% | – | 0 | – | 1.08 (0.50–1.66) | 12 | 95.8% |
|  | CK | 1.09 (0.57–1.61) | 5 | 86.4% | 0.88 (0.14–1.62) | 2 | 89.9% | – | 0 | – | 1.03 (0.62–1.43) | 7 | 86.6% |
|  | D-dimer | 0.82 (0.35–1.28) | 8 | 90.7% | 0.69 (0.46–0.93) | 2 | 0.0% | – | 0 | – | 0.79 (0.44–1.15) | 10 | 88.3% |
|  | Creatinine | 0.82 (0.39–1.26) | 8 | 86.8% | 0.66 (0.43–0.89) | 3 | 0.0% | – | 0 | – | 0.78 (0.46–1.11) | 11 | 83.1% |
|  | Total bilirubin | 0.43 (0.04–0.83) | 5 | 77.6% | 0.18 (-0.15–0.50) | 1 | – | – | 0 | – | 0.39 (0.05–0.72) | 6 | 76.5% |
|  | ALT | 0.39 (0.11–0.68) | 7 | 67.0% | 0.23 (-0.02–0.62) | 2 | 48.8% | – | 0 | – | 0.37 (0.15–0.58) | 9 | 61.0% |
|  | PT | 0.23 (-0.16–0.61) | 4 | 69.7% | – | 0 | – | – | 0 | – | 0.23 (-0.16–0.61) | 4 | 69.7% |
|  | Hemoglobin | 0.02 (-0.25–0.30) | 4 | 50.8% | -0.32 (-0.65–-0.001) | 3 | 40.7% | – | 0 | – | -0.11 (-0.35–0.14) | 7 | 63.0% |
|  | Monocyte count | -0.18 (-1.05–0.69) | 3 | 92.2% | – | 0 | – | – | 0 | – | -0.18 (-1.05–0.69) | 3 | 92.2% |
|  | Platelet count | -0.62 (-0.96–-0.28) | 5 | 71.5% | -0.04 (-0.27–0.18) | 3 | 0.0% | – | 0 | – | -0.42 (-0.75–-0.10) | 8 | 80.4% |
|  | Lymphocyte count | -0.64 (-0.90–-0.39) | 9 | 70.4% | -0.30 (-0.91–0.32) | 3 | 81.7% | – | 0 | – | -0.58 (-0.80–-0.35) | 12 | 72.5% |

Numbers are presented as pooled risk ratios or pooled standardized mean difference unless specified otherwise. The numbers under the column name "n" represents the number of studies included in the meta-analysis in the previous column, and I^2^ represents the degree of heterogeneity.

Abbreviations: WMD, weighted mean difference; LDH, lactate dehydrogenase; BUN, blood urea nitrogen; AST, aspartate transaminase; CK, creatine kinase; WBC, white blood cell; ALT, alanine transaminase; PT, prothrombin time.

^*^Includes both troponin I and troponin T.
